# Accuracy of Continuous Glucose Monitoring During Three Closed-Loop Home Studies Under Free-Living Conditions

**DOI:** 10.1089/dia.2015.0062

**Published:** 2015-11-01

**Authors:** Hood Thabit, Lalantha Leelarathna, Malgorzata E. Wilinska, Daniella Elleri, Janet M. Allen, Alexandra Lubina-Solomon, Emma Walkinshaw, Marietta Stadler, Pratik Choudhary, Julia K. Mader, Sibylle Dellweg, Carsten Benesch, Thomas R. Pieber, Sabine Arnolds, Simon R. Heller, Stephanie A. Amiel, David Dunger, Mark L. Evans, Roman Hovorka

**Affiliations:** ^1^ Wellcome Trust-MRC Institute of Metabolic Science, University of Cambridge, Cambridge, United Kingdom.; ^2^ Academic Unit of Diabetes, Endocrinology and Metabolism, Department of Human Metabolism, University of Sheffield, Sheffield, United Kingdom.; ^3^ Diabetes Research Group, King's College London, London, United Kingdom.; ^4^ Division of Endocrinology and Metabolism, Department of Internal Medicine, Medical University of Graz, Graz, Austria.; ^5^ Profil, Neuss, Germany.

## Abstract

***Objectives:*** Closed-loop (CL) systems modulate insulin delivery based on glucose levels measured by a continuous glucose monitor (CGM). Accuracy of the CGM affects CL performance and safety. We evaluated the accuracy of the Freestyle Navigator^®^ II CGM (Abbott Diabetes Care, Alameda, CA) during three unsupervised, randomized, open-label, crossover home CL studies.

***Materials and Methods:*** Paired CGM and capillary glucose values (10,597 pairs) were collected from 57 participants with type 1 diabetes (41 adults [mean±SD age, 39±12 years; mean±SD hemoglobin A1c, 7.9±0.8%] recruited at five centers and 16 adolescents [mean±SD age, 15.6±3.6 years; mean±SD hemoglobin A1c, 8.1±0.8%] recruited at two centers). Numerical accuracy was assessed by absolute relative difference (ARD) and International Organization for Standardization (ISO) 15197:2013 15/15% limits, and clinical accuracy was assessed by Clarke error grid analysis.

***Results:*** Total duration of sensor use was 2,002 days (48,052 h). Overall sensor accuracy for the capillary glucose range (1.1–27.8 mmol/L) showed mean±SD and median (interquartile range) ARD of 14.2±15.5% and 10.0% (4.5%, 18.4%), respectively. Lowest mean ARD was observed in the hyperglycemic range (9.8±8.8%). Over 95% of pairs were in combined Clarke error grid Zones A and B (A, 80.1%, B, 16.2%). Overall, 70.0% of the sensor readings satisfied ISO criteria. Mean ARD was consistent (12.3%; 95% of the values fall within ±3.7%) and not different between participants (*P*=0.06) within the euglycemic and hyperglycemic range, when CL is actively modulating insulin delivery.

***Conclusions:*** Consistent accuracy of the CGM within the euglycemic–hyperglycemic range using the Freestyle Navigator II was observed and supports its use in home CL studies. Our results may contribute toward establishing normative CGM performance criteria for unsupervised home use of CL.

## Introduction

Intensive insulin therapy is advocated to reduce the risk of complications in type 1 diabetes, but hypoglycemia remains a significant barrier to tightening glycemic control.^1^ There is currently an unmet need to reduce the burden of diabetes care on patients and healthcare providers. Closed-loop (CL) systems, which use a control algorithm to direct insulin delivery autonomously in a glucose-responsive manner by coupling a real-time subcutaneous continuous glucose monitor (CGM) and subcutaneous insulin delivery by a pump, may transform the management of type 1 diabetes.^2^ Over the past decade, CL research has made incremental progress from studies performed in clinical research facility and transitional settings to the home environment.^3–5^ Unsupervised home studies provide the ultimate test bed for CL systems and reflect daily life conditions without intervention or monitoring by researchers. As modulation of insulin delivery is based on CGM sensor glucose levels, sensor performance is important in determining the efficacy and safety of CL systems.

Accuracy of commercially available CGM devices has improved over the years. Glucose sensor accuracy studies in ambulatory patients at home have reported mean absolute relative difference (MARD), a statistical measure of the difference between two related measures, of around 12–19%.^6,7^ At least 80% of measurements from current CGM devices lie within Zone A and B, deemed clinically accurate or benign errors, of the Clarke error grid (CEG) analysis.^8^ However, glucose sensor accuracy in the hypoglycemic range as assessed by MARD is known to be worse compared with during euglycemia.^9^ There are few published reports of CGM performance during CL studies. Unsupervised crossover home studies provide a unique opportunity to assess accuracy of real-time CGM across all glycemic ranges during standard therapy and CL insulin delivery in an environment reflecting the user's free-living home conditions.

The present study assessed the accuracy of the Freestyle Navigator^®^ II real-time CGM (Abbott Diabetes Care, Alameda, CA) during two overnight^10,11^ and one day-and-night^12^ unsupervised CL home studies under free-living conditions.

## Research Design and Methods

### Participants

Forty-one adults (18 females, 23 males) and 16 adolescents (six females, 10 males) with type 1 diabetes were recruited as part of CL home studies in these two cohorts. All participants were on insulin pump therapy for at least 3 months prior to joining these studies. Adult participants were identified from eligible patients attending Addenbrooke's Hospital (Cambridge, United Kingdom), Sheffield Teaching Hospitals (Sheffield, United Kingdom), King's College Hospital (London, United Kingdom), Medical University of Graz (Graz, Austria), and Profil Institute (Neuss, Germany). Adolescent participants were enrolled through the Paediatric Diabetes Clinics at Addenbrooke's Hospital and University College London Hospital.

### Study design and procedures

Details of the CL study design and experimental protocols have been described previously.^10–12^ In summary, following a 2–4-week run-in phase, participants used insulin pump therapy with real-time continuous glucose monitoring at home for two periods with or without CL. Each study period lasted 4 (adults) or 3 (adolescents) weeks in the overnight CL studies and 1 week in the day-and-night CL study.^10–12^ Identical study insulin pump and real-time CGMs were used during the two study periods, which were separated by a 3–4-week washout period, during which participants used their own pump and discontinued continuous glucose monitoring.

Participants were not restricted in dietary intake or daily activities during either CL or control periods. No supervision or telemonitoring was provided throughout the study. A 24-h telephone support service was available for any clinical or technical issues that arose during the study. User manuals and troubleshooting literature for the CGM were provided to all participants.

All participants/guardians provided written informed consent and assent, as appropriate. The study protocol was approved by the respective research ethics and regulatory committees in the United Kingdom, Germany, and Austria.

### Continuous glucose monitoring system

The Freestyle Navigator II real-time CGM system^13^ was used in all three CL home studies. Participants were instructed on the insertion and use of the continuous glucose monitoring system following enrollment into the study. During the CL and control periods, participants were instructed to calibrate the sensor according to the manufacturer's instructions using the built-in Freestyle glucose meter. In addition, during the CL period participants were advised to recalibrate the sensor before going to bed (overnight and day-and-night CL use) or in the morning (day-and-night CL use) if the sensor was over-reading by more than 3.0 mmol/L. This advice mitigated the risk of sensor error leading to insulin overdelivery during CL operation as assessed by in silico testing^14^ using the validated Cambridge simulator.^15^ Participants were advised to wash their hands before calibration to reduce measurement errors. Each Freestyle glucose meter was checked using calibration fluid prior to the start of the study.

### Numerical and clinical accuracy

CGM sensor accuracy was evaluated using data collected during the open-loop and CL periods but not during run-in or washout periods. Numerical accuracy was assessed by absolute deviation, absolute relative difference (ARD), and using the International Organization for Standardization (ISO) 15197:2013 15/15% limits for acceptability of capillary home glucose monitoring meters^16^ (95% of the measured glucose values should fall within either ±0.83 mmol/L [15 mg/dL] of the average measured values of the reference measurement procedure at a glucose level of <5.55 mmol/L or within 15% at a glucose level of >5.55 mmol/L). Main measured outcomes included overall MARD and median ARD, as well as MARD and median ARD in the euglycemic (3.9–10.0 mmol/L), hypoglycemic (<3.9 mmol/L), and hyperglycemic (>10.0 mmol/L) ranges stratified according to capillary glucose measurements. A Bland–Altman analysis plot was used to compare the limits of agreement between real-time continuous glucose sensor and capillary glucose values. The percentage of data points of the CEG was used to evaluate clinical accuracy.

### Statistical analyses

Statistical analyses were performed using SPSS version 21 software (IBM Software, Portsmouth, United Kingdom). Data are presented as mean (SD) or median (interquartile range) values unless stated otherwise. Demographics and clinical characteristic variables in the first and fourth quartiles were compared using the independent-samples *t* test for normally distributed data or the Mann–Whitney U test for non-normally distributed variables. MARD was compared between treatments and participants by repeated-measures analysis of variance to account for correlated data using participant-level per-treatment MARD in the analysis. Difference in MARD between participants was tested by analysis of variance, and the null hypothesis was accepted if the variance of random subject effect was not different from zero.

## Results

CGM sensor data from 57 participants were analyzed: 41 adults (mean±SD age, 39±12 years; mean±SD hemoglobin A1c, 7.9±0.8%) and 16 adolescents (mean±SD age, 15.6±3.6 years; mean±SD hemoglobin A1c, 8.1±0.8%). Baseline demographics of participants are outlined in Table 1. The average number of fingerstick capillary glucose measurements performed by adult participants was 5.9±2.0 per day, and that performed by adolescent participants was 4.3±2.4 per day.

**Table 1.** Baseline Demographics of Participants from the Three Closed-Loop Home Studies

	*Adults (*n*=41)*	*Adolescents (*n*=16)*
Age (years)	39.5±11.6	15.4±2.0
Gender (male/female)	23/18	10/6
Body mass index (kg/m^2^)	26.1±3.4	21.7±2.2
Hemoglobin A1c (%)	7.9±0.8	8.1±0.9
Duration of diabetes (years)	24.8±11.6	7.2±4.3

### Sensor numerical accuracy

**FIG. 1.**
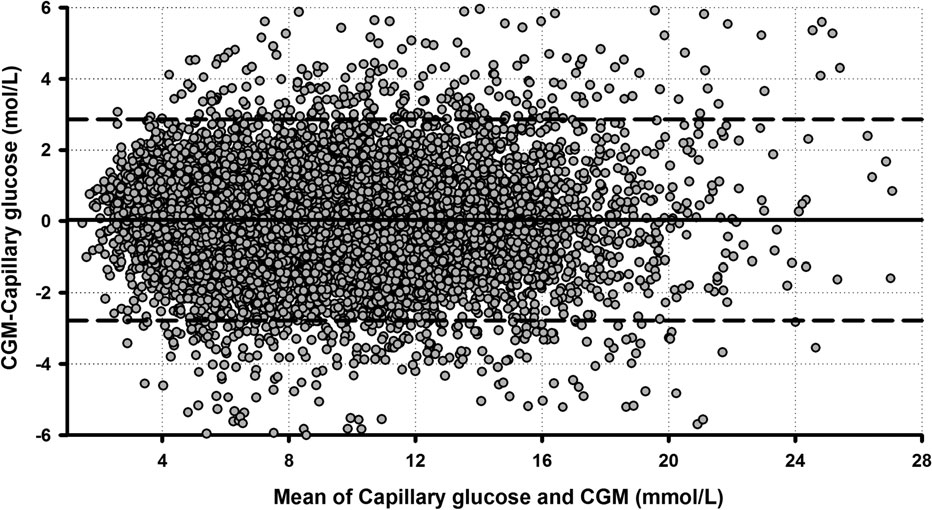
Bland–Altman plot of sensor and capillary glucose levels. The solid black line represents the mean difference between the sensor and capillary glucose values; the dashed lines indicate 1.96×SD of the difference. CGM, continuous glucose monitor.

The total duration of sensor use for the whole cohort was 2,002 days (48,052 h), equivalent to 90% of total study duration (53,424 h). Duration of sensor use per week was higher in the adult compared with the adolescent cohort (155.8 [148.7, 162.2] h vs. 149.7 [143.2, 152.4] h; *P*=0.033). In total, 10,647 sensor–capillary glucose pairs were available for the analysis (Table 2). The overall numerical sensor accuracy expressed as MARD and median ARD was 14.2±15.5% and 10.0% (4.5%, 18.4%), respectively. The MARD in the euglycemia range was 13.9±13.3% (*n*=6,223 pairs). The Freestyle Navigator II CGM accuracy as measured by ARD was higher in the hyperglycemic range (MARD, 9.8±8.8%; *n*=3,410 pairs). In the hypoglycemic range (*n*=1,014 pairs), MARD and median ARD were 30.6±28.7% and 22.8% (11.2%, 41.2%), respectively. Information related to sensor accuracy over the range of glucose values defined is shown in the Bland–Altman plot in Figure 1. The distribution of participant-level MARD over the whole capillary glucose range and above 3.9 mmol/L shows that a greater proportion of participants achieved MARD below 15% in the euglycemia and hyperglycemia range (Supplementary Fig. S1; Supplementary Data are available online at www.liebertonline.com/dia). Overall, 70.0% of the Freestyle Navigator II sensor readings were within ISO 15197:2013 15/15% accuracy criteria (<3.9 mmol/L, 55.7%; 3.9–10.0 mmol/L, 67.5%; and >10.0 mmol/L, 78.7%).

**Table 2.** Sensor Accuracy for the Whole Glucose Range and Stratified According to Capillary Glucose During the Three Closed-Loop Home Studies

	*Whole range (1.1–27.8 mmol/L)*	*Euglycemia (3.9–10.0 mmol/L)*	*Hyperglycemia (>10.0 mmol/L)*	*Hypoglycemia (<3.9 mmol/L)*
Number of glucose sensor–capillary pairs	10,647	6,223	3,410	1,014
Mean capillary glucose (mmol/L)	8.6±3.9	6.9±1.7	13.3±2.8	3.2±0.5
Mean AD	1.0±1.0	0.9±0.8	1.3±1.2	0.9±0.8
Median AD	0.8 (0.4, 1.4)	0.7 (0.3, 1.2)	1.0 (0.4, 1.8)	0.7 (0.4, 1.2)
Mean ARD	14.2±15.5	13.9±13.3	9.8±8.8	30.6±28.7
Median ARD (%)	10.0 (4.5, 18.4)	10.4 (4.7, 18.6)	7.8 (3.4, 13.7)	22.8 (11.2, 41.2)
Bias	0.04 (1.44)	0.08 (1.3)	−0.25 (1.7)	0.7 (0.9)
ISO criteria (%)^a^	70.0	67.5	78.7	55.7

Data are mean±SD or median (interquartile range) values as indicated.

^a^ International Organization for Standardization (ISO) 15197:2013 15/15% limits.

AD, absolute difference; ARD, absolute relative difference.

MARD values per participant during CL and control periods were comparable for the whole cohort (13.9±15.0% vs. 14.4±16.0%; *P*=0.13). MARD was higher in adults compared with adolescents (14.5±16.0% vs. 13.0±13.8%; *P*<0.001). Across the whole glucose range, MARD differences between participants was found to be statistically significant (13.9%; 95% of the values fall within ±4.1%; *P*=0.001); however, MARD between participants was more comparable when excluding capillary glucose values at or below 3.9 mmol/L from calculating MARD (12.3%; 95% of the values fall within ±3.7%; *P*=0.06). The demographics and clinical characteristics of participants (age, body mass index, duration of diabetes, HbA1c, mean glucose level, SD of glucose level, and percentage of time spent <3.9 mmol/L) with the lowest sensor accuracy (MARD in the worst fourth quartile) were compared against those with the highest accuracy (MARD in the best first quartile) (Supplementary Table S1). Proportion of time spent in the hypoglycemia range was significantly higher in participants with the lowest sensor accuracy (4.9% [2.8%, 8.5%] vs. 2.2% [1.5%, 5.2%]; *P*=0.032). No other significant findings were observed. Reciprocally, when first and fourth quartiles of participants' demographics and clinical characteristics were compared, sensor accuracy was significantly lower (MARD, 15.6% [13.4%, 16.9%] vs. 12.2% [11.0%, 13.3%]; *P*=0.01) in those spending most time in the hypoglycemia range (Supplementary Table S2).

### Sensor clinical accuracy

**FIG. 2.**
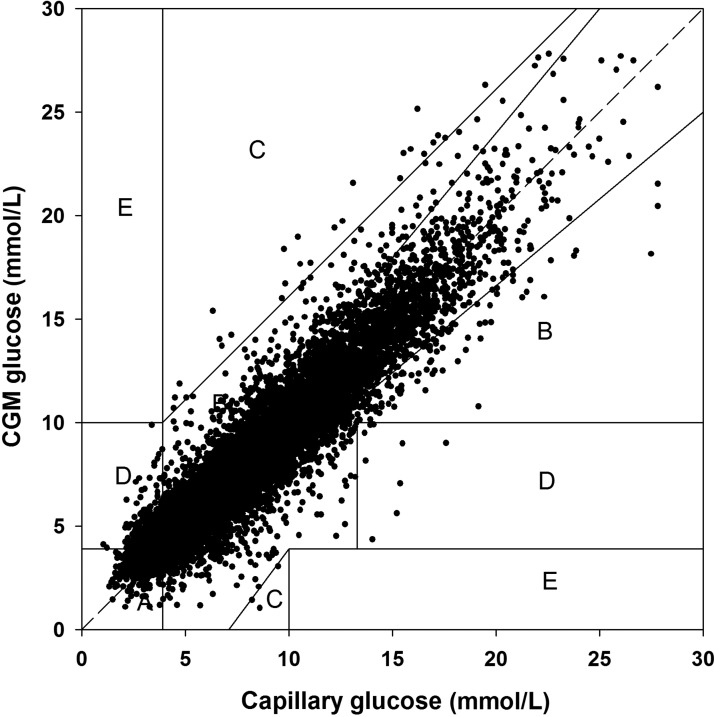
Clarke error grid of sensor and capillary glucose levels. CGM, continuous glucose monitor.

The Freestyle Navigator CGM II had 96.3% of measurements in CEG Zones A+B (Zone A, 80.1%; Zone B, 16.2%; Zone C, 0.2%; Zone D, 3.5%; Zone E, 0.0%) (Fig. 2). During the CL and control periods, 96.5% were in CEG Zones A+B (Zone A, 80.3%; Zone B, 16.2%; Zone C, 0.2%; Zone D, 3.3%; Zone E, 0.0%), and 96.1% were in CEG Zones A+B (Zone A, 79.9%; Zone B, 16.2%; Zone C, 0.3%; Zone D, 3.7%; Zone E, 0.0%), respectively.

## Discussion

We investigated the numerical and clinical accuracy of the Freestyle Navigator II CGM during three CL home studies under free-living conditions. Our analysis suggests that in ambulatory real-life conditions, the Freestyle Navigator II CGM achieved nearly comparable MARD scores with those reported in the manufacturer's labeling and in controlled research facility settings.^9,17^ The reliability and consistency of CGM performance are particularly important in CL home studies. As expected and shown in other studies, the Freestyle Navigator II CGM was less accurate in the hypoglycemic range compared with the euglycemic range when measured by ARD (MARD, 30.6% vs. 13.9%), but absolute difference was comparable (mean, 0.9 vs. 0.9 mmol/L). Nonetheless, the accuracy measured by MARD was higher in the hyperglycemic range, and the Bland–Altman plot demonstrated relatively few variations in sensor accuracy over the range of measured glucose concentrations. Sensor accuracy was not found to be significantly different between participants in the euglycemic and hyperglycemic range, when insulin delivery is actively modulated by the control algorithm. From a CL perspective, this facilitates safer operation of CL by avoiding hypoglycemia resulting from sensor over-reading, whereas this risk is mitigated in the hypoglycemia range as insulin delivery is suspended by the algorithm. The clinical relevance of the accuracy analysis is further substantiated by the percentage values in Zone A and B of the CEG analysis, suggesting that over 95% of the input data to the control algorithm from CGM had led to either clinically appropriate decisions or avoided inappropriate treatment.

Previously published CGM accuracy studies were mostly performed in clinical research facility settings.^17,18^ Experimental conditions in inpatient studies do not fully reflect free-living conditions, although such studies benefit from gold-standard reference glucose measurements. Some studies do not assess CGM performance across all glycemia ranges.^19,20^ An inpatient head-to-head comparison study reported superior overall accuracy for the Navigator CGM, when compared with the Dexcom^®^ Seven^®^ Plus (Dexcom, Inc., San Diego, CA; the latter superseded by Dexcom G4™ Platinum) and the Guardian^®^ (Medtronic, Northridge, CA) (now superseded by the Enlite™ sensor).^8^ When paired with venous glucose measured by the GlucoScout™ (International Biomedical, Austin, TX), the percentage points in Zone A of the CEG were comparable to our analysis (80.6%). Another recent inpatient study compared the Navigator, Dexcom G4 Platinum, and Enlite CGM sensors.^17^ Using venous glucose as reference, the Navigator and Dexcom G4 Platinum had the best overall accuracy (MARD, 12.3% and 10.8%, respectively).

Direct comparability to assess CGM performance during home use of CL can be challenging, as inpatient studies commonly use plasma or venous glucose as reference measurements or for calibration. Such conditions are not practical in routine clinical practice, and participants would normally rely on capillary glucose measurements. Based on the limited number of published reports on CGM performance in the home environment, data from our analysis are comparable. Kropff et al.^7^ compared the accuracy of the Dexcom G4 Platinum and Medtronic Enlite CGM system during 6 days of home use. The overall accuracy in the home setting was better for the Dexcom G4 Platinum compared with the Enlite (MARD, 12.2±12.0% vs. 19.9±20.5%; *P*<0.0001).

These findings were complemented by another ambulatory head-to-head CGM home comparison study by Matuleviciene et al.^6^ The accuracy of a modified Dexcom G4 Platinum system with an enhanced calibration algorithm was recently assessed over 7 days.^21^ The MARD during home use was 11.3%, with 92.4% in CEG Zone A. These studies were of a shorter duration and had fewer numbers of CGM–capillary glucose reference pairs available for analysis, especially in the hypoglycemia range. To our knowledge, no other CL home studies of similar duration have published data related to CGM accuracy and performance.

The incidence and duration of large sensor errors are other important determinants that may impact on the efficacy and safety of a CL system. Although a continuous error grid analysis has been specifically developed to evaluate CGM performance,^22^ such a measurement tool has yet to be used widely and does not provide information about the duration, severity, and incidence of large sensor errors. A recent study quantifying the incidence and duration of large inaccuracy found that the Freestyle Navigator was safer for CL use compared with the Dexcom Seven Plus.^23^ Although the Dexcom Seven Plus has been superseded by Dexcom G4 Platinum with comparable performance to FreeStyle Navigator in terms of large sensor errors,^24^ the report highlights the unmet need of having additional measures to assess CGM performance and reliability during CL operation.

Determining individual-level factors associated with reduced accuracy would help identify strategies to optimize sensor performance. Post hoc analysis of our results revealed that individuals with the most time spent in the hypoglycemia range had lower MARD overall. This is consistent with findings of lower MARD in the hypoglycemia range and may potentially contribute to bias in studies reporting primarily on hypoglycemia reduction outcomes. Further work is needed to determine individual-level factors influencing sensor performance and impact on CL performance.

The strengths of the present analysis are real-world settings and the large dataset spanning a broad age range and geographical locations. We assessed sensor accuracy across a wide glycemic range experienced by participants during free-living conditions. Capillary glucose data used in the analysis were obtained directly from the capillary blood glucose meter built-in within the Freestyle Navigator CGM, thereby eliminating timing and transcription errors and standardizing device use. The limitations include pre-analytical errors associated with fingerstick capillary glucose testing at home, which may lead to erroneous measurements and calibration. However, participants were instructed to perform hand washing before performing fingerstick testing as per usual clinical practice.

In summary, we observed high and consistent numerical and clinical accuracy of the Freestyle Navigator II CGM in the nonhypoglycemic range during home use in CL studies during free-living conditions. In the hyperglycemic range, the improved numerical accuracy facilitates safe operation of CL by avoiding hypoglycemia from CGM sensor over-reading. Conversely, sensor inaccuracy at the hypoglycemic range is mitigated by insulin delivery suspension during CL operation. Our analysis supports use of the Freestyle Navigator in unsupervised CL home studies and may contribute toward establishing CGM performance criteria for home use of CL systems.

## Supplementary Material

Supplemental data

Supplemental data

Supplemental data
