# Supplementary material for: Accuracy of Continuous Glucose Monitoring During Three Closed-Loop Home Studies Under Free-Living Conditions
Source: Diabetes Technol Ther. 2015 Nov 1;17(11):801–7. doi: 10.1089/dia.2015.0062 (PMC4649721; doi:10.1089/dia.2015.0062)
Supplement: Supplemental data [file Supp_Fig1.pdf]

Supplementary Data

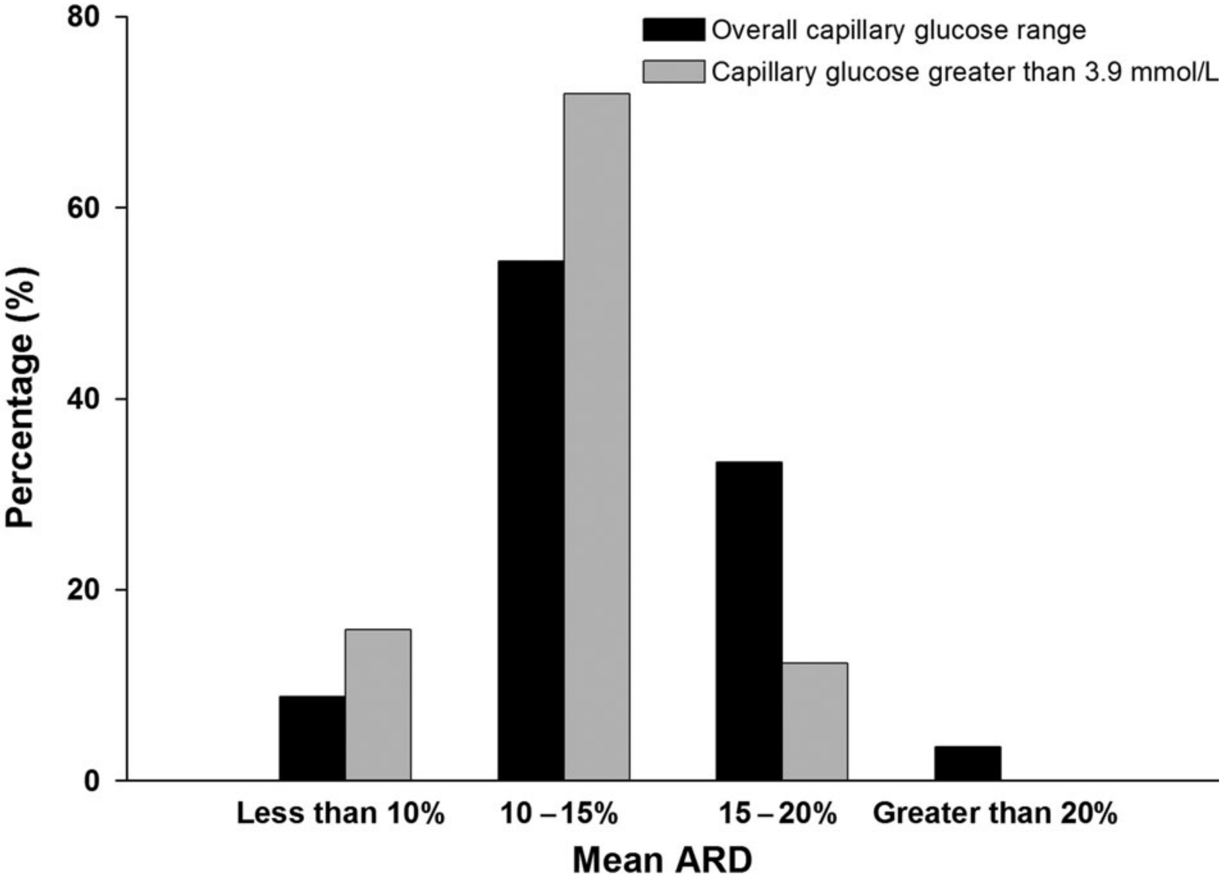

SUPPLEMENTARY FIG. S1. Distribution of mean absolute relative difference (ARD) among participants ($n = 57$). The plot shows the percentage of participants with participant-level mean ARD within the given ranges. The participant-level mean ARD is calculated using sensor–capillary glucose pairs across the whole glucose range (black bars) and in non-hypoglycemia (gray bars).
