# Supplementary material for: Accuracy of Continuous Glucose Monitoring During Three Closed-Loop Home Studies Under Free-Living Conditions
Source: Diabetes Technol Ther. 2015 Nov 1;17(11):801–7. doi: 10.1089/dia.2015.0062 (PMC4649721; doi:10.1089/dia.2015.0062)
Supplement: Supplemental data [file Supp_Table1.pdf]

Supplementary Table S1. Clinical Characteristics and Demographics of Participants According to Sensor Accuracy (Mean Absolute Relative Difference) Quartiles Q1–Q4

	MARD (%) quartiles			
	Q1 (8.51–11.95)	*Q2 (11.96–13.61)*	*Q3 (13.62–16.13)*	*Q4 (16.14–21.85)*
Age (years)	34.6 (16.7, 42.9)	45.1 (33.5, 48.8)	20.1 (16.5, 34.2)	31.8 (27.0, 38.9)
BMI (kg/m^2)	25.3 ± 4.6	25.0 ± 3.9	23.8 ± 3.1	25.3 ± 3.0
Duration of diabetes (years)	10.0 (4.8, 28.0)	21.5 (10.0, 39.0)	13.2 (7.2, 20.3)	20.9 (17, 24.5)
HbA1c (%)	8.2 ± 1.1	7.8 ± 0.9	7.8 ± 0.6	7.8 ± 0.7
Mean glucose (mmol/L)	8.7 ± 1.5	8.9 ± 1.0	8.6 ± 0.8	8.6 ± 0.6
SD of sensor glucose (mmol/L)	3.0 ± 0.5	2.8 ± 0.5	3.0 ± 0.3	3.3 ± 0.5
Proportion of time spent <3.9 mmol/L (%)	2.2 (1.5, 5.2)	1.7 (1.1, 2.2)	2.9 (1.9, 6.1)	4.9 (2.8, 8.5)

Data are mean ± SD or median (interquartile range) values as indicated.
BMI, body mass index; HbA1c, hemoglobin A1c; MARD, mean absolute relative difference; Q, quartile.
