# Supplementary material for: Accuracy of Continuous Glucose Monitoring During Three Closed-Loop Home Studies Under Free-Living Conditions
Source: Diabetes Technol Ther. 2015 Nov 1;17(11):801–7. doi: 10.1089/dia.2015.0062 (PMC4649721; doi:10.1089/dia.2015.0062)
Supplement: Supplemental data [file Supp_Table2.pdf]

Supplementary Table S2. Comparison of Mean Absolute Relative Difference According to Clinical Characteristics and Demographics Data Divided into Quartiles Q1–Q4

	MARD (%)
Age (years)	
Q1 (12.3–17.9)	12.2 (10.5, 13.7)
Q2 (18.0–33.0)	16.7 (15.6, 17.2)
Q3 (33.5–42.9)	13.2 (11.0, 14.8)
Q4 (43.1–68.8)	12.5 (12.1, 13.6)
BMI (kg/m^2)	
Q1 (18.0–21.8)	13.2 ± 2.9
Q2 (22.0–24.6)	15.2 ± 3.4
Q3 (25.0–27.5)	14.7 ± 2.6
Q4 (27.8–34.6)	12.6 ± 2.7
Duration of diabetes (years)	
Q1 (1.7–9.2)	12.1 (9.9, 14.5)
Q2 (9.8–18.0)	13.7 (12.5, 16.8)
Q3 (19.2–28)	14.8 (12.0, 17.0)
Q4 (28.7–56.0)	12.9 (11.6, 14.8)
HbA1c (%)	
Q1 (6.1–7.4)	14.2 ± 3.1
Q2 (7.5–7.9)	14.4 ± 3.1
Q3 (8.0–8.6)	14.3 ± 3.0
Q4 (8.7–9.6)	12.2 ± 2.5
Mean glucose (mmol/L)	
Q1 (6.6–8.0)	13.0 ± 3.3
Q2 (8.1–8.7)	16.1 ± 2.8
Q3 (8.8–9.5)	14.1 ± 2.6
Q4 (9.6–10.8)	12.5 ± 1.9
SD of sensor glucose (mmol/L)	
Q1 (1.9–2.7)	13.5 ± 3.1
Q2 (2.8–3.0)	12.9 ± 2.0
Q3 (3.1–3.4)	14.0 ± 3.3
Q4 (3.5–4.2)	15.3 ± 3.2
Proportion of time spent <3.9 mmol/L (%)	
Q1 (0.3–1.7)	12.2 (11.0, 13.3)
Q2 (1.7–2.4)	12.5 (12.0, 16.1)
Q3 (2.8–5.2)	15.1 (12.2, 17.0)
Q4 (5.7–13.2)	15.6 (13.4, 16.9)

Data are mean ± SD or median (interquartile range) as indicated.

BMI, body mass index; HbA1c, hemoglobin A1c; MARD, mean absolute relative difference; Q, quartile.
